# The environmental health literacy level was effectively improved of residents in Shaanxi Province, China, 2022

**DOI:** 10.3389/fpubh.2024.1499349

**Published:** 2025-01-13

**Authors:** Feidan Deng, Xinyue Wen, Guoqiang Dong, Xining Wang, Huifang He, Ruixuan Zhu, Lichun Qiao, Jing Han

**Affiliations:** ^1^Institute for Hygiene of Ordnance Industry, Xi'an, China; ^2^School of Public Health, Xi'an Jiaotong University Health Science Center, Xi'an, China; ^3^Key Laboratory for Disease Prevention and Control and Health Promotion of Shaanxi Province, Xi'an, China

**Keywords:** environmental health literacy (EHL), influencing factors, Shaanxi Province, urban–rural gap, health education

## Abstract

**Background:**

Environmental health literacy (EHL) aims to enable individuals to make informed choices to reduce health risks and protect the environment.

**Objectives:**

To assess the EHL of residents in Shaanxi Province, China in 2022 and analyze the influencing factors.

**Methods:**

This study was a 2022 cross-sectional survey in Shaanxi Province, China, involving 2,237 residents aged 15 to 69. Participants were selected using a multi-stage random sampling method and surveyed through questionnaires in six cities. The weighted rate was calculated using the seventh National Census data, and influential factors were analyzed using multifactor logistic regression.

**Results:**

According to the survey, the overall EHL level of Shaanxi residents in 2022 was 15.47%. The first-level classification literacy level of EHL was ranked in descending order: basic skills (21.64%), basic concepts (17.93%), and basic knowledge (14.44%). The EHL level was influenced by age, education level, and occupation. People with higher education and certain occupations were more likely to possess EHL, while older individuals and those with lower education levels, especially in rural areas, were less likely to possess it.

**Conclusion:**

The level of EHL among residents in Shaanxi Province in 2022 was higher than in 2020. The findings showed that targeted health education for low EHL groups effectively reduced urban–rural disparities and improved adolescents’ EHL levels.

## 1 Introduction

Environmental health is an expanding field of knowledge, constantly growing and refining the evidence base that connects the environment with human health (1). Environmental factors that create health risks include water (including sanitation and good hygiene), air pollution, noise, chemical and radiation exposure, recreational risks (e.g., drowning), risks associated with land-use practices (including risks from buildings), and other risks related to the workplace or community and climate change (2). Environmental pollution impacts health through emissions from multiple sources, contamination across different media, and exposure through various pathways, leading to significant and often irreversible risks (3). According to an analysis by WHO in 2016, 24% of deaths globally (and 28% of deaths in children under five) were attributable to modifiable environmental factors (4). Therefore, it is essential for people to raise awareness of the relationship between the environment and health and to integrate environmental protection into the health protection system.

In 2008, the American Association for Public Health Education introduced the new term “environmental health literacy (EHL)” for the first time globally, defining it as an individual’s ability to search for, locate, understand, and recognize the environmental and health information, and using that information to make good choices to reduce health risks, improve quality of life, and protect the environment (5). EHL is an emerging multidisciplinary field that integrates the concepts of environmental and health literacy, develops professional knowledge and awareness, enables people to understand and use information appropriately, and promotes behavioral change, which positively impacts individual and public health and the environment (6, 7).

Currently, there are relatively few studies on EHL at home and abroad (8). Several foreign studies have explored the relationship between EHL and race, ethnicity, and socioeconomic status, as well as household air pollution (6, 9, 10). In 2018, 15 provinces conducted the first EHL survey of residents, resulting in an EHL of 12.5% for Chinese residents (11). In 2019, the Healthy China Initiative (2019–2030) included a goal to reach an EHL level of 25% for the Chinese population by 2030 (12). As the public health risks from environmental pollution due to China’s industrialization and urbanization will gradually increase, it is crucial to enhance the population’s EHL and promote overall health (13).

This study aims to conduct a comprehensive survey on the level of EHL of the residents from Shaanxi Province, China. According to the requirements of the Technical Guidelines for Measuring Citizens’ Environmental and Health Literacy (for Trial Implementation), to assess the EHL level and its influencing factors. The goal is to provide targeted suggestions for improving the EHL level and to offer insights for other countries or regions to enhance their EHL levels (14).

## 2 Materials and methods

### 2.1 Study design

The study is a cross-sectional survey conducted in Shaanxi Province, China, in 2022. It focused on residents aged 15 to 69 years who lived in six district cities within the Shaanxi Province, including Xi’an, Weinan, Yulin, Yan’an, Hanzhong, and Ankang. Participants had resided in these areas for a consecutive six-month period.

**Figure 1:**
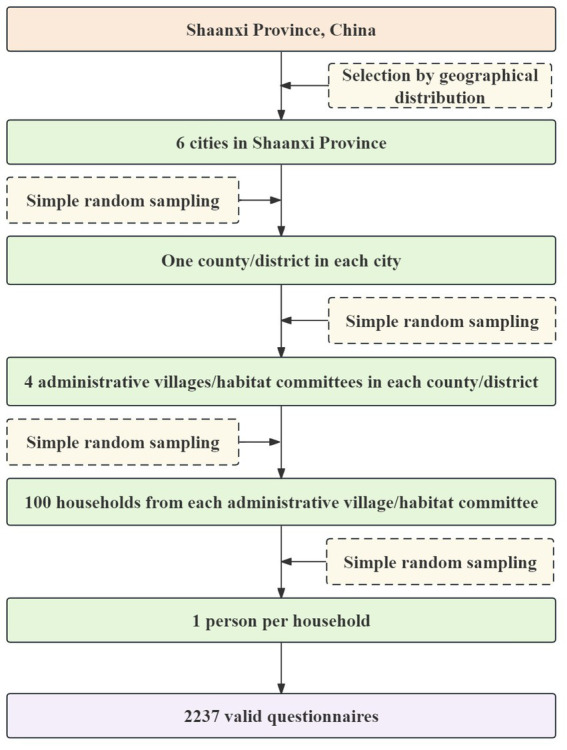
Flowchart for sampling participants in the 2022 environmental health literacy study for residents aged 15–69 in Shaanxi Province, China.

A multi-stage random sampling method was employed to select participants in this study, and Figure 1 illustrates the sampling process. The minimum sample size for each stratum was calculated using Equation 1:


(1) $$n=\left(Z^{2}p\left(1–p\right)\right)e^{2}\times \mathrm{deff}$$

*n* is the minimum sample size per stratum; *z* is usually set at 1.96; *p* is the previous level of EHL, the value of *p* is 0.13; *e* is the absolute permissible error, *e* = p*relative error (the relative error is usually set 10–20%); *deff* is the value of the random effect of the complex design, which is usually set at 1.5–2.0. The total sample size was estimated as follows by Equation 2:


(2) $$\begin{array}{l} N=n\left(n_{FPC}\right)\times \left(\mathrm{Product of stratification factors}\right)\times \\ \left(1+\mathrm{failure rate}\right)\text{.} \end{array}$$

*N* is the total sample size; *n* or *nFPC* is the minimum sample size for each stratum or the minimum sample size corrected for a finite population. The product of stratification factors: stratification factors were chosen based on demographic and sociological characteristics that can impact the level of EHL and the purpose of the assessment. For instance, if we choose 2 strata for urban and rural areas and 2 strata for gender, then the product of the stratification factors will be 2 × 2 = 4 strata. The failure to visit rate is set at 5 to 10%.

To account for cases where participants could not be reached or refused to participate, a rate of 10% was calculated for lost visits and refusal. To ensure enough participants, each neighborhood committee or administrative village added 10 eligible households as a backup to the 100 original households. The final number of households included in the analysis was 2,237.

### 2.2 Questionnaire survey

**Figure 2:**
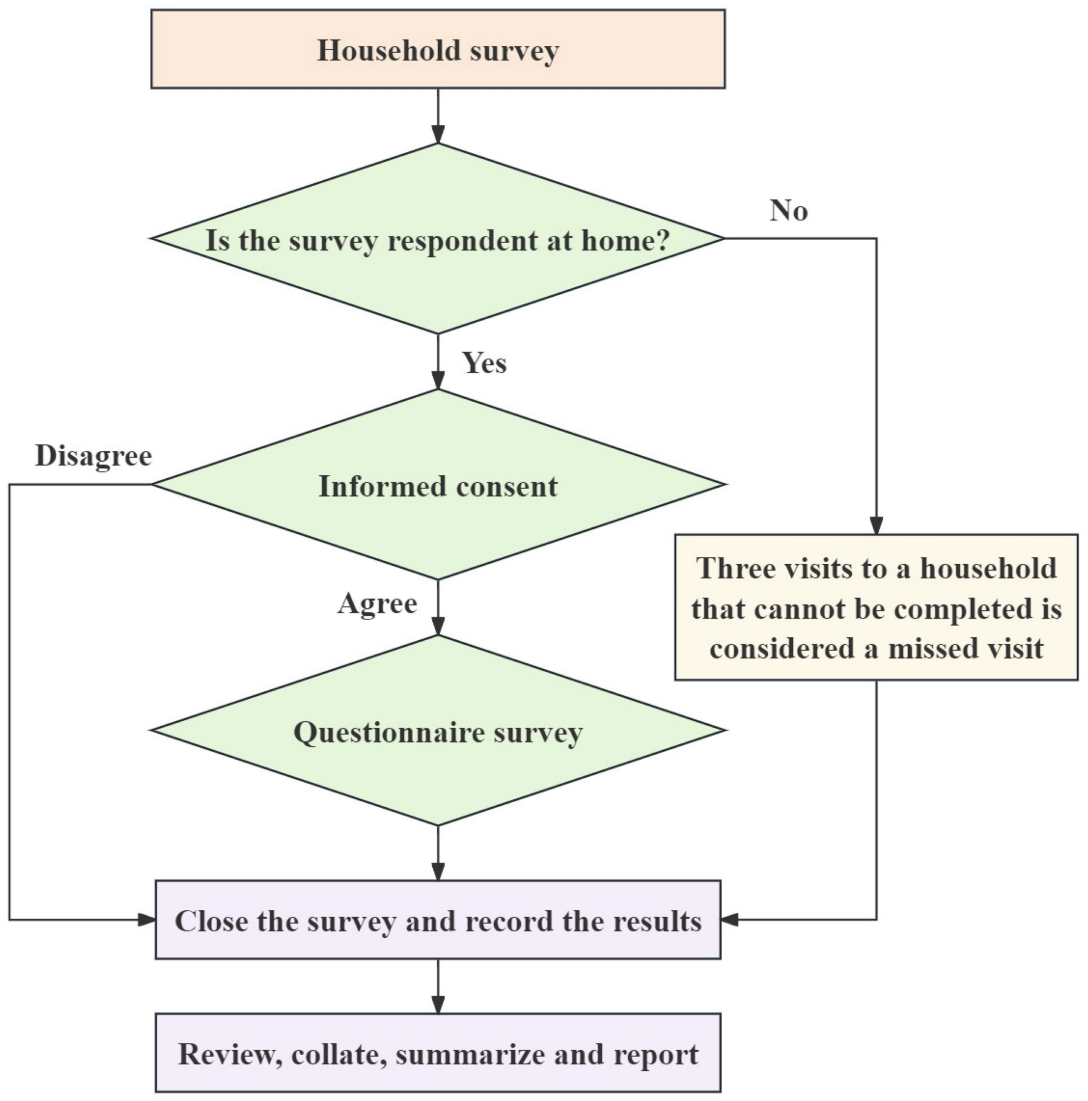
Flowchart of the household survey in the 2022 environmental health literacy survey for the population aged 15–69 years in Shaanxi Province, China.

All participants completed a written informed consent form before the study. Investigators, who had undergone uniform training and completed an assessment, collected the questionnaires in households. The household survey process is shown in Figure 2.

The Environmental and Health Literacy Assessment Questionnaire consists of 47 questions, divided into three categories: 13 judgmental questions, 14 single-choice questions, and 20 multiple-choice questions. The questionnaire covered three main categories: basic concepts, basic knowledge, and basic skills. These categories were further divided into basic cognition, basic attitude, fundamental concept, scientific knowledge, basic behavior, and basic skills. The basic concepts section covers understanding environmental pollution hazards, health risks, and the responsibility to prevent risks and protect the environment. The basic knowledge section includes information on air, water, soil, household garbage, environmental health, and toxic and hazardous substances. The basic skills section includes green and healthy lifestyles, emergency response, and monitoring skills.

The KMO (Kaiser-Meyer-Olkin) value of the questionnaire was 0.940, and Bartlett’s test of sphericity was statistically significant with a *p*-value of less than 0.001. The questionnaire showed good internal consistency, with a reliability score of 0.887 and a Cronbach’s alpha coefficient of 0.885 (15).

### 2.3 Score criteria

The questionnaire has a total score of 100 points. The scoring system is as follows: for each correct answer to a judgment question, you receive 1 point, while each wrong answer earns 0 points. For single-choice questions, each correct answer is worth 2 points, and each incorrect answer earns 0 points. For multiple choice questions, selecting the correct answer earns 3 points, while an incorrect or missed answer earns 0 points. If individuals’ total score equals or exceeds 70, they will be categorized as having EHL. The level of EHL is the percentage of people with EHL within the total population under observation.

### 2.4 Statistical analysis

The results of the questionnaire were analyzed using SPSS version 26 (IBM, Armonk, NY, United States). Categorical variables were expressed as frequencies (percentages). The chi-square test was employed to compare categorical variables in different groups. The weighted rate was calculated using data from the seventh National Census (16), and influential factors were analyzed using multifactor logistic regression. A difference was deemed statistically significant when the *p*-value was less than 0.05 (two-tailed). *p*-values in bold indicated statistical significance. *p < 0.05, **p < 0.01, ***p < 0.001.

## 3 Results

### 3.1 Demographic characteristics and EHL status

The demographic characteristics and EHL status of the 2,237 respondents are in Table 1. The mean age of the respondents was 42.78 ± 15.13 years, with 51.68% as male and 47.61% as urban residents. The age distribution was as follows: 31.69% were aged 15–34, 31.56% were aged 35–49, and 36.75% were aged 50–69. Additionally, 85.07% of the respondents held a junior high school degree or above. Among the respondents, 48.73% were farmers, 22.53% were urban workers, and 14.66% were students.

**Table 1:** Demographic characteristics and level of environmental health literacy (EHL) in the participants (*n* = 2,237).

Variables	Category	Number of survey	Level of EHL			*X* ^2^	*p*
			n	Rate (%)	Weighted rate (%)		
Gender	Male	1,156	172	14.88	13.20	0.633	0.426
	Female	1,081	174	16.10	13.97		
Age groups	15–34	709	208	29.34	23.90	179.168	**< 0.001** ^***^
	35–49	706	100	14.16	13.25		
	50–69	822	38	4.62	4.00		
Region	Urban	1,065	162	15.21	13.76	0.102	0.750
	Rural	1,172	184	15.70	13.23		
Education level	Primary school and below	334	7	2.10	0.94	250.120	**< 0.001** ^***^
	Junior high school	853	62	7.27	6.26		
	Senior high school / vocational / technical secondary school	539	95	17.63	13.46		
	Junior college/bachelor degree and above	511	182	35.62	30.29		
Occupation	Urban workers	504	78	15.48	12.88	216.122	**< 0.001** ^***^
	Teacher	53	13	24.53	22.19		
	Leading cadres and civil servants	107	48	44.86	34.78		
	Peasantry	1,090	83	7.61	6.11		
	Student	328	110	33.54	29.69		
	The emeritus and retired	71	2	2.82	6.03		
	Others	84	12	14.29	12.54		
Environmental protection worker	Yes	118	22	18.64	13.60	0.962	0.327
	No	2,119	324	15.29	12.87		
Total		2,237	346	15.47	15.25		

346 respondents in Shaanxi Province had an EHL score of at least 70, reflecting an overall EHL level of 15.47%. After weighted adjustment of the data, the overall level of EHL of residents in Shaanxi Province was 15.25%. The study found significantly higher EHL levels in the 15–34 age group and among respondents with higher education. In the occupational classifications, leading cadres and civil servants (44.86%) had the highest EHL levels, followed by students (33.54%) and teachers (24.53%). Farmers and retired participants had the lowest EHL levels. There were no significant differences in EHL levels between genders, urban and rural areas, and whether or not individuals were practitioners involved in ecological and environmental protection.

**Figure 3:**
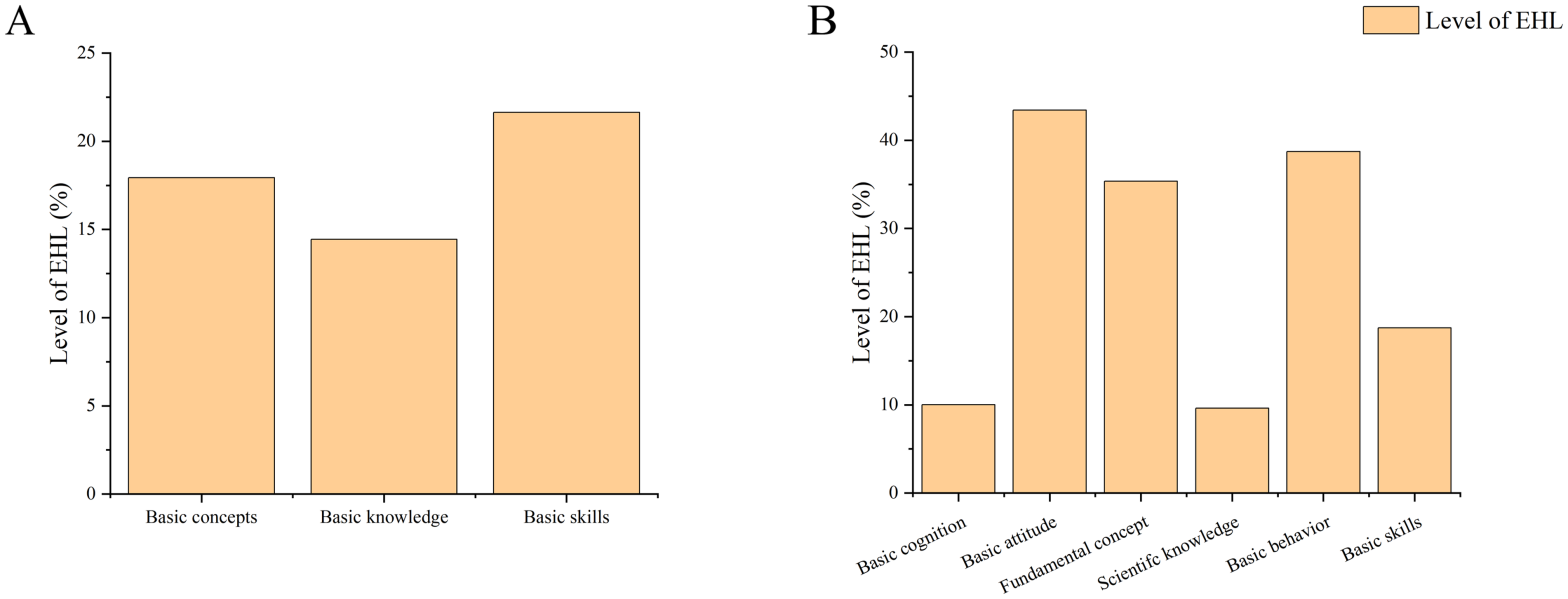
Overall level of first-level and second-level classification literacy. **(A)** The first-level classification literacy; **(B)** the second-level classification literacy.

The first-level classification literacy of EHL of residents was ranked as follows: basic skills (21.64%), basic concepts (17.93%), and basic knowledge (14.44%; Figure 3A). There was a noticeable lack of fundamental understanding of environmental and health matters among residents. The second-level classification literacy of EHL of residents was: basic cognition (10.01%), basic attitude (43.41%), fundamental concept (35.36%), scientific knowledge (9.61%), basic behavior (38.71%), and basic skills (38.71%; Figure 3B). The deficiency in basic cognition and scientific knowledge was especially noticeable. Residents demonstrated the highest accuracy in basic attitudes related to environmental and health issues, but the lowest correct rate of scientific knowledge about the health effects of air, water, and other environmental pollution.

### 3.2 Relationship between EHL score and first-level classification literacy

The statistical analysis employed linear regression with EHL scores as the dependent variable and basic concepts, basic knowledge, and basic skills as independent variables. The regression model demonstrated a F statistic of 1420.972 (*p* < 0.001) and an adjusted R-squared of 0.656. These findings suggest that the influence of the three first-level classification literacy on the total EHL score was ranked in the following order: basic knowledge (0.418) > basic skills (0.348) > basic concepts (0.280; Table 2).

**Table 2:** The linear regression relationship between the total score of EHL and first-level classification literacy.

Dimensions	Unstandardized coefficients	Standardized coefficients	t	*p*
Constant	−0.021		−3.945	< 0.001^***^
Basic concepts	0.264	0.280	20.051	**< 0.001** ^***^
Basic knowledge	0.430	0.418	29.542	**< 0.001** ^***^
Basic skills	0.306	0.348	24.783	**< 0.001** ^***^

### 3.3 Stratified analysis of first-level classification literacy

**Figure 4:**
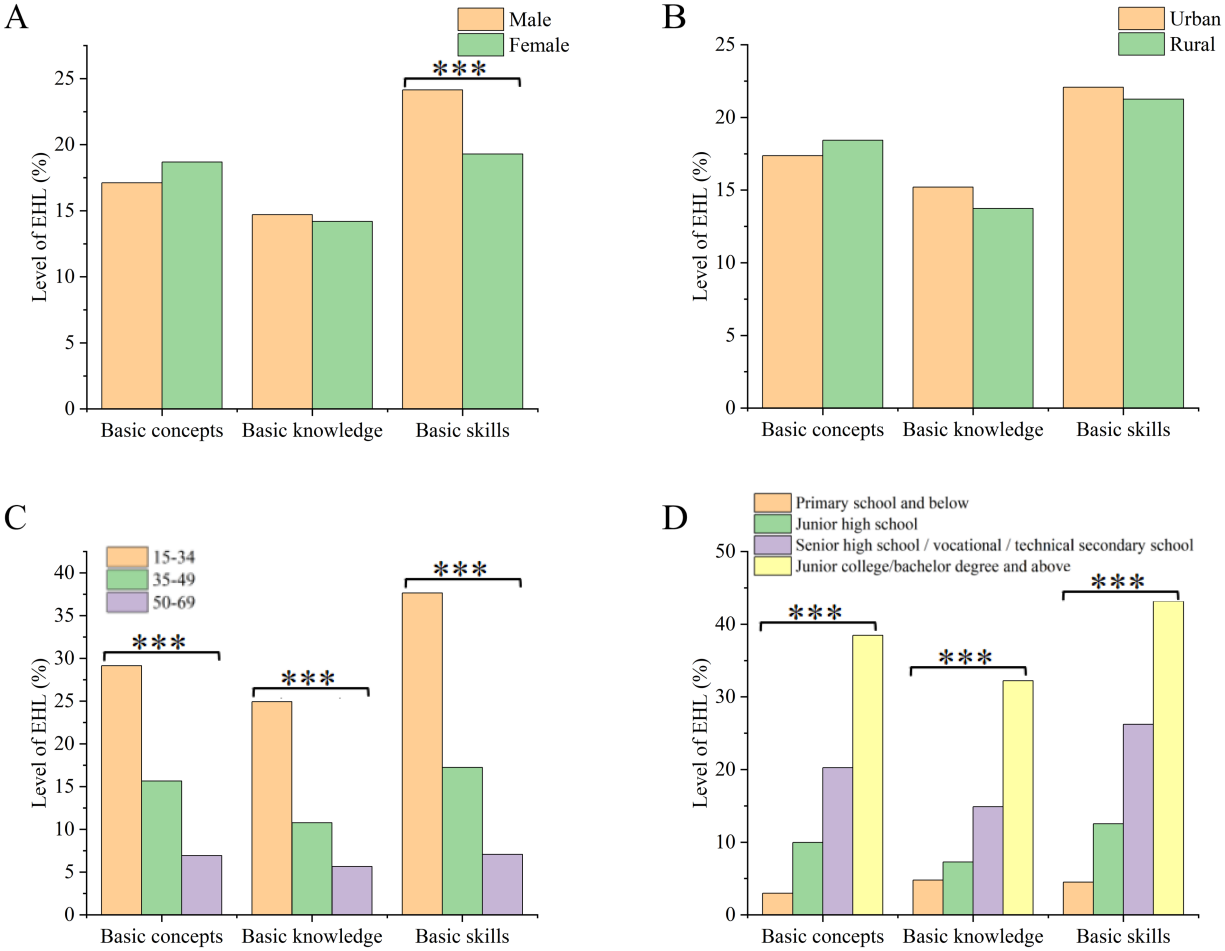
Stratified analysis of first-level classification literacy. **(A)** Stratified by gender; **(B)** Stratified by region; **(C)** Stratified by age; **(D)** Stratified by educational level.

The stratified analysis of the first-level classification literacy of EHL level (Figure 4) showed that males had slightly higher scores than females in basic skills. However, there was no gender difference in basic concepts and basic knowledge (Figure 4A). Additionally, there was no significant difference in the first-level classification literacy between urban and rural residents (Figure 4B). The 15–34 age group has the highest first-level classification literacy across all three dimensions, followed by the 35–49 and 50–69 age groups (Figure 4C). The understanding of first-level classifications improves with higher education levels. Those with junior college/bachelor’s degrees and above have the highest first-level classification literacy at 35.55% (Figure 4D).

### 3.4 Stratified analysis of second-level classification literacy

**Figure 5:**
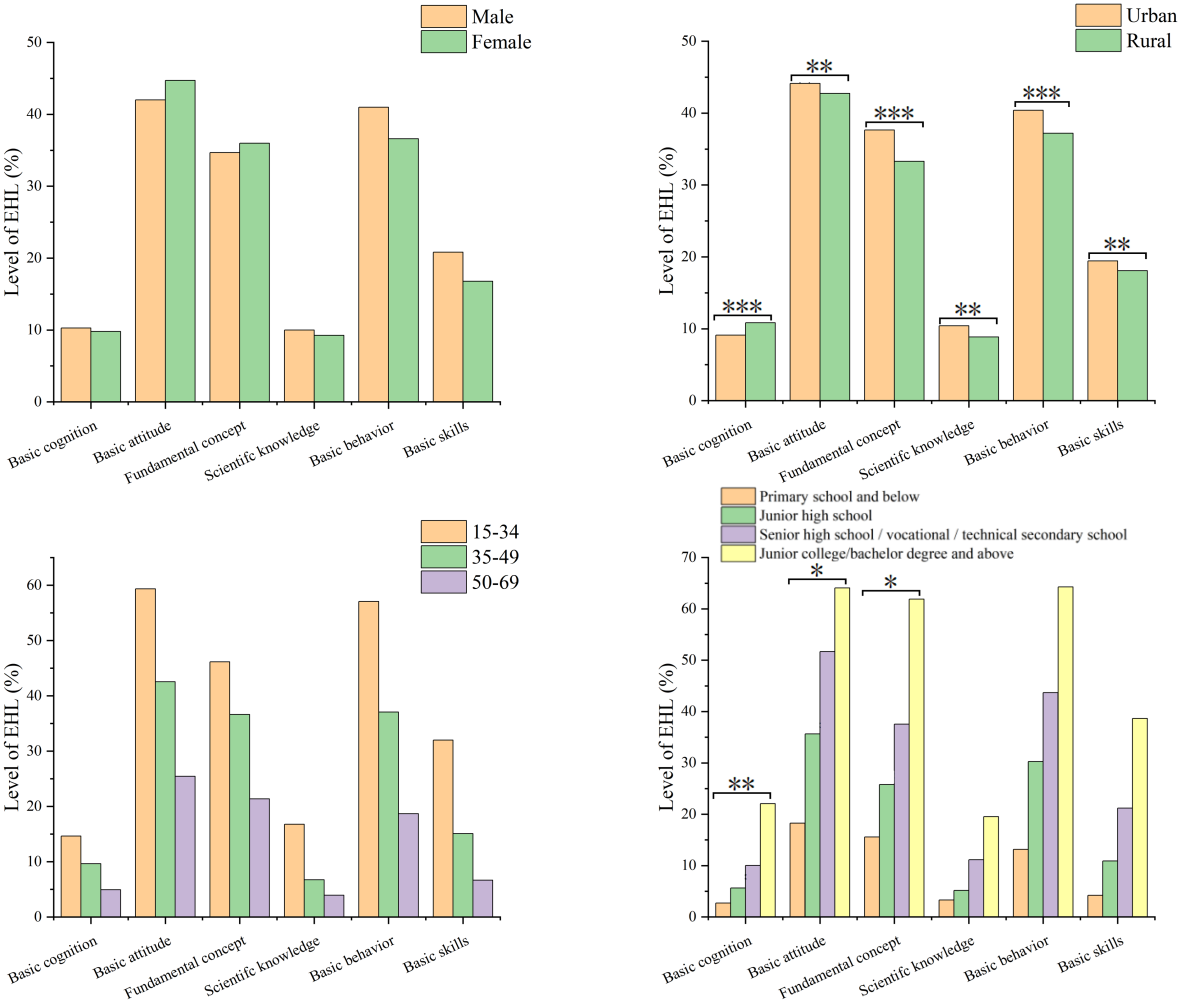
Stratified analysis of second-level classification literacy. **(A)** Stratified by gender; **(B)** Stratified by region; **(C)** Stratified by age; **(D)** Stratified by educational level.

The stratified analysis of second-level classification literacy of EHL level (Figure 5) showed no gender disparity (*p* > 0.05; Figure 5A). Apart from basic cognition, urban residents demonstrated a higher level of second-level classification literacy compared to rural residents (*p* < 0.05; Figure 5B). There were no statistically significant variances in the six dimensions of second-level classification literacy across different age groups (*p* > 0.05) (Figure 5C). The level of second-level classification literacy showed varying degrees of improvement with higher levels of education (Figure 5D).

### 3.5 The influencing factors of EHL

In binary logistic regression, gender, age, education level, and occupation were included, and Odds ratios (OR) and 95% confidence intervals (95% CI) were calculated by overall and region, respectively (Table 3). Overall, the 35–49 age groups [OR = 0.679, 95% CI (0.485 to 0.950)] and 50–69 age groups [OR = 0.387, 95% CI (0.245 to 0.611)] were less likely to possess EHL than 15–34 age groups. Notably, education level was positively correlated with EHL possession. Furthermore, the research shows that leading cadres and civil servants [OR = 2.698, 95% CI (1.661 to 4.383)] and students [OR = 2.129, 95% CI (1.434 to 3.162)] are more likely to possess EHL than urban workers.

**Table 3:** Odds ratios (OR) and 95% confidence intervals of having basic EHL stratified by region in the enter logistic regression.

Risk factor	Overall		Urban		Rural	
	OR (95% CI)	*p*	OR (95% CI)	*p*	OR (95% CI)	*p*
Gender						
Male	Reference		Reference		Reference	
Female	0.953 (0.739, 1.230)	0.713	1.103 (0.758, 1.604)	0.609	0.856 (0.601, 1.220)	0.390
Age groups						
15–34	Reference		Reference		Reference	
35–49	0.679 (0.485, 0.950)	**<0.05** ^*^	0.959 (0.570, 1.613)	<0.874	0.533 (0.337, 0.843)	**<0.01** ^**^
50–69	0.387 (0.245, 0.611)	**<0.001** ^***^	0.575 (0.285, 1.161)	<0.123	0.284 (0.154, 0.524)	**<0.001** ^***^
Education level						
Primary school and below	Reference		Reference		Reference	
Junior high school	2.336 (1.039, 5.249)	**<0.05** ^*^	4.797 (0.632, 36.383)	0.129	2.051 (0.827, 5.087)	0.121
Senior high school/vocational/ technical secondary school	5.489 (2.439, 12.356)	**<0.001** ^***^	14.675 (1.952, 110.305)	**<0.01** ^**^	3.935 (1.561, 9.920)	**<0.01** ^**^
Junior college/bachelor degree and above	11.187 (4.894, 25.573)	**<0.001** ^***^	35.618 (4.718, 268.913)	**<0.01** ^**^	5.890 (2.235, 15.520)	**<0.001** ^***^
Occupation						
Urban workers	Reference		Reference		Reference	
Teacher	0.846 (0.422, 1.696)	0.637	1.231 (0.357, 4.250)	0.742	0.830 (0.346, 1.991)	0.676
Leading cadres and civil servants	2.698 (1.661, 4.383)	**<0.001** ^***^	1.303 (0.524, 3.243)	0.569	3.932 (2.092, 7.389)	**<0.001** ^***^
Peasantry	1.038 (0.718, 1.500)	0.843	1.039 (0.611, 1.766)	0.887	0.988 (0.588, 1.660)	0.962
Student	2.129 (1.434, 3.162)	**<0.001** ^***^	3.366 (1.892, 5.988)	**<0.001** ^***^	1.367 (0.775, 2.410)	0.280
The emeritus and retired	0.339 (0.077, 1.484)	0.151	0.530 (0.110, 2.548)	0.428	-	0.998
Others	0.886 (0.449, 1.749)	0.727	0.715 (0.257, 1.987)	0.519	1.035 (0.411, 2.608)	0.941

Stratified by region (Table 3), respondents with higher educational levels among urban and rural residents showed a higher likelihood of possessing an EHL. Furthermore, residents aged 35–49 years [OR = 0.533, 95% CI (0.337 to 0.843)] and 50–69 years [OR = 0.284, 95% CI (0.154 to 0.524)] in rural areas were less inclined to possess EHL. For occupational classification, students [OR = 3.366, 95% CI (1.892 to 5.988)] among urban residents and leading cadres and civil servants [OR = 3.932, 95% CI (2.092 to 7.389)] among rural residents were more likely to possess EHL.

Stratified by gender, age, and educational level (Supplementary Tables S1–S3), the results showed that higher education was associated with higher EHL levels, but higher age was associated with lower EHL levels. Residents of occupations such as leading cadres and civil servants, students, and teachers were more likely to possess EHL. Interestingly, among junior high school and below residents, peasantry [OR = 4.262, 95% CI (1.341, 13.547)] showed a higher likelihood of possessing an EHL than Urban workers.

## 4 Discussion

In this study, we assessed the EHL level of residents in Shaanxi Province in 2022. We investigated the factors influencing EHL overall and stratified by gender, age, region, and education level. After the weighted adjustment, the overall EHL level of residents in Shaanxi province was 15.25%, higher than the 12.95% EHL level in 2020, reaching the target value of the Healthy China Action (2019–2030) (17). The findings showed that to enhance the EHL of residents in Shaanxi Province, it is essential to focus on middle-aged and older adult individuals, farmers, and retirees, while improving overall education levels. Additionally, providing targeted health education on environmental and health knowledge, basic cognition, and scientific knowledge could help improve the EHL of residents.

The EHL level in Shaanxi in 2022 increased by 19.3% compared to 2020, which was higher than the 15% required by the Healthy China Initiative (2019–2030) in 2022 (17), but still far from the national overall average of 18.8% (18). In 2022, the EHL level of residents in Shaanxi Province was lower than that of Guangxi Province (22.46%) (19), Guangdong Province (18.82%) (20), and Hubei Province (18.2%) (21). Therefore, Shaanxi Province still needs to continue to make efforts to improve the EHL of residents. In this study, there was no significant difference in EHL levels between rural and urban residents (15.21% vs. 15.70%). It is inconsistent with the EHL survey of Chinese residents (22) (8.1% vs. 16.9%), the 2020 survey results of Shaanxi Province (23) (25.00% vs. 11.51%), and the 2022 survey results of Guangdong Province (20), etc. The possible reason is that Shaanxi Province has implemented several measures to improve the EHL of rural residents based on the 2020 survey results, which indicated a low EHL among the rural population. These measures included initiating a pilot project to promote EHL among rural residents, displaying informational materials in administrative villages, distributing posters, brochures, and promotional videos, and organizing thematic exhibitions. These measures have been proven to have a positive impact and can be applied more broadly (24).

The study found that participants in the 15–34 age group had the highest EHL levels. Additionally, EHL levels decreased with age in both men and women, particularly in the 50–69 age group. These findings are consistent with surveys conducted in Jiangsu Province (25) and Guangxi Province (26). The possible reason is that residents aged 50–69 have a relatively low level of education, slow acceptance of new knowledge, and lack of active learning awareness, so the EHL level of residents will be low. The 15–34 age group had the highest EHL levels, which may be due to the fact that residents in this age group are young people, have a wide range of access to knowledge, and have strong cognitive abilities (27). At the same time, in response to the 2020 findings indicating low EHL among adolescents and children, Shaanxi Province has established the “Shaanxi Provincial Youth Ecological Environment and Health Literacy Promotion Base.” They conducted a series of science activities to create a harmonious ecological environment and share a green and healthy life.

In this study, higher education levels were associated with higher levels of EHL, as well as first-level and second-level classification literacy. It was consistent with the findings of Jiangsu Province (25), Guangxi Province (28), and Hubei Province (29). Thus, the level of EHL is closely related to the educational level. Residents who were leading cadres or civil servants, as well as teachers and students, had a higher level of EHL than other occupations. It may be related to the nature of their work, and they have advantages in the way and ability to acquire new knowledge (25).

Residents in Shaanxi Province lacked basic knowledge of the environment and health, and the low level of basic cognition and scientific knowledge literacy was prominent. It was consistent with the findings of numerous studies involving medical students (30), college students (31), and community residents (20, 28). It can be seen that the low awareness rate of basic knowledge, basic cognition, and scientific knowledge was a common problem among different groups and regions. According to the results of linear regression analysis, the impact of first-level classification literacy on the total EHL score was as follows: basic knowledge > basic skills > basic concepts. The overall mastery rate of basic knowledge was only 14.44%. The low level of basic knowledge is the direct cause of the low level of EHL. Knowledge is at the forefront of the “Know-Believe-Act” chain, and the lack of basic knowledge and scientific knowledge will inevitably affect the formation of basic concepts and the acquisition of basic skills (29, 32). Therefore, the government and the community should attach great importance to health education, especially to strengthen the basic knowledge and scientific knowledge of the ecological environment and health.

Binary logistic regression results show that age, education level, and occupation significantly influence residents’ EHL. EHL level decreases with age and increases with education level. People with lower education levels, older age groups, farmers, and retirees tend to have relatively low EHL levels. According to the results of this study, to effectively improve the EHL level of residents in Shaanxi Province, the following suggestions are put forward: Firstly, the low levels of basic and scientific knowledge in EHL significantly impact overall EHL. It is necessary to increase investment in popular science, strengthen the creation of popular science on environmental health, and promote the sharing of works and resources. For example, inviting experts and celebrities to produce popular science videos and establishing environmental health sections on platforms like WeChat and TikTok to disseminate basic and scientific knowledge closely related to residents’ daily lives (33). Secondly, set up expert and student volunteer teams to conduct widespread environmental health science outreach in communities, rural areas, campuses, and enterprises. Use varied communication methods to educate the older adults and farmers about the health benefits of EHL (34, 35). Thirdly, the knowledge of EHL should be incorporated into education, as education plays a crucial role in determining the EHL of residents. It is vital to focus on educating adolescents and children, utilizing digital technology to create educational materials with diverse and engaging content to enhance the EHL of minors and ultimately raise the overall EHL level in Shaanxi province.

Our research emphasizes the need for targeted health education programs for populations with lower health literacy, such as seniors, farmers, and retirees. These programs can empower individuals to make informed health and environmental choices. Additionally, it is crucial to carry out educational initiatives that combine environmental protection with health promotion to encourage proactive behavioral changes. Future studies should assess the long-term effects of these programs, explore EHL’s impact on health outcomes, and consider how various factors shape EHL to tailor effective, equitable health education strategies.

### 4.1 Limitations

Firstly, the survey conducted in the six cities covered a limited area, and due to the impact of the COVID-19 pandemic on our research process, we were unable to collect information from 100 households in some administrative villages, resulting in fewer than the anticipated 2,400 participants. However, our entire sampling process strictly adhered to the principles of multi-stage random sampling, and the one-on-one field research was conducted strictly according to the execution guidelines. Therefore, our study maintains good representativeness for the residents of Shaanxi Province. Secondly, the data in this study was obtained from a cross-sectional survey, which limits the interpretation of the results and makes it difficult to draw general conclusions.

## 5 Conclusion

The level of EHL among residents in Shaanxi Province increased in 2022 compared to 2020. Following the 2020 survey results, Shaanxi Province implemented various health education programs aimed at residents in rural areas and adolescents, which have yielded positive outcomes. The results showed that customized health education for groups with low EHL effectively narrowed the gap between urban and rural areas, enhancing the EHL levels of adolescents.

## Data Availability

The original contributions presented in the study are included in the article/Supplementary material, further inquiries can be directed to the corresponding authors.
