# Supplementary material for: The environmental health literacy level was effectively improved of residents in Shaanxi Province, China, 2022
Source: Front Public Health. 2025 Jan 13;12:1499349. doi: 10.3389/fpubh.2024.1499349 (PMC11769948; doi:10.3389/fpubh.2024.1499349)
Supplement: Supplementary file 1 [file Table_1.DOCX]

Table S1 Odds ratios (OR) and 95% confidence intervals of having basic EHL stratified by gender in the enter logistic regression

| **Risk factor** | **Male** | |  | **Female** | |
| --- | --- | --- | --- | --- | --- |
|  | **OR (95% CI)** | ***P*** |  | **OR (95% CI)** | ***P*** |
| Age groups |  |  |  |  |  |
| 15-34 | Reference |  |  | Reference |  |
| 35-49 | **0.509 (0.312, 0.829)** | **<0.01^**^** |  | **0.942 (0.583, 1.521)** | **0.806** |
| 50-69 | **0.370 (0.191, 0.717)** | **<0.01^**^** |  | **0.423 (0.222, 0.806)** | **<0.01^**^** |
| Region |  |  |  |  |  |
| Urban | Reference |  |  | Reference |  |
| Rural | **1.225 (0.844, 1.779)** | **0.286** |  | **0.973 (0.678, 1.396)** | **0.882** |
| Education level |  |  |  |  |  |
| Primary school and below | Reference |  |  | Reference |  |
| Junior high school | **1.458 (0.573, 3.713)** | **0.429** |  | **7.406 (0.991, 55.365)** | **0.051** |
| Senior high school/vocational/ technical secondary school | **3.497 (1.363, 8.970)** | **<0.01^**^** |  | **18.026 (2.414, 134.626)** | **<0.01^**^** |
| Junior college/bachelor degree and above | **8.476 (3.185, 22.554)** | **<0.001^***^** |  | **32.364 (4.297, 243.736)** | **<0.01^**^** |
| Occupation |  |  |  |  |  |
| Urban workers | Reference |  |  | Reference |  |
| Teacher | **1.202 (0.520, 2.778)** | **0.667** |  | **0.369 (0.078, 1.748)** | **0.209** |
| Leading cadres and civil servants | **3.143 (1.425, 6.933)** | **<0.01^**^** |  | **2.192 (1.177, 4.083)** | **<0.05^*^** |
| Peasantry | **1.414 (0.808, 2.472)** | **0.225** |  | **0.830 (0.502, 1.373)** | **0.468** |
| Student | **3.806 (2.083, 6.957)** | **<0.001^***^** |  | **1.385 (0.806, 2.381)** | **0.238** |
| The emeritus and retired | **0.431 (0.053, 3.523)** | **0.432** |  | **0.302 (0.037, 2.429)** | **0.260** |
| Others | **1.298 (0.533, 3.160)** | **0.566** |  | **0.554 (0.180, 1.705)** | **0.303** |

Table S2 Odds ratios (OR) and 95% confidence intervals of having basic EHL stratified by age in the enter logistic regression

| **Risk factor** | **15-34** | |  | **35-49** | |  | **50-69** | |
| --- | --- | --- | --- | --- | --- | --- | --- | --- |
|  | **OR (95% CI)** | ***P*** |  | **OR (95% CI)** | ***P*** |  | **OR (95% CI)** | ***P*** |
| Gender |  |  |  |  |  |  |  |  |
| Male | Reference |  |  | Reference |  |  | Reference |  |
| Female | **0.683 (0.481, 0.970)** | **<0.05^*^** |  | **1.741 (1.093, 2.773)** | **<0.05^*^** |  | **0.960 (0.491, 1.878)** | **0.904** |
| Region |  |  |  |  |  |  |  |  |
| Urban | Reference |  |  | Reference |  |  | Reference |  |
| Rural | **0.963 (0.676, 1.371)** | **0.834** |  | **1.269 (0.797, 2.022)** | **0.315** |  | **1.073 (0.546, 2.109)** | **0.839** |
| Education level |  |  |  |  |  |  |  |  |
| Primary school and below | Reference |  |  | Reference |  |  | Reference |  |
| Junior high school | **0.883 (0.101, 7.740)** | **0.911** |  | **3.549 (0.454, 27.770)** | **0.228** |  | **2.492 (0.904, 6.874)** | **0.078** |
| Senior high school / vocational /technical secondary school | **1.678 (0.194, 14.503)** | **0.638** |  | **18.253 (2.420, 137.691)** | **<0.01^**^** |  | **4.089 (1.351, 12.379)** | **<0.05^*^** |
| Junior college/bachelor degree and above | **3.331 (0.387, 28.688)** | **0.273** |  | **36.916 (4.861, 280.371)** | **<0.001^***^** |  | **6.682 (1.326, 33.669)** | **<0.05^*^** |
| Occupation |  |  |  |  |  |  |  |  |
| Urban workers | Reference |  |  | Reference |  |  | Reference |  |
| Teacher | **1.287 (0.039, 3.072)** | **0.570** |  | **0.271 (0.058, 1.267)** | **0.097** |  | **-** | **-** |
| Leading cadres and civil servants | **3.920 (1.782, 8.620)** | **<0.001^***^** |  | **1.452 (0.715, 2.945)** | **0.302** |  | **8.857 (1.628, 48.180)** | **<0.05^*^** |
| Peasantry | **0.752 (0.403, 1.403)** | **0.370** |  | **1.095 (0.624, 1.922)** | **0.751** |  | **2.575 (0.753, 8.800)** | **0.131** |
| Student | **2.099 (1.334, 3.303)** | **<0.001^***^** |  | **0.972 (0.384, 2.464)** | **0.953** |  | **0.951 (0.150, 6.019)** | **0.957** |
| The emeritus and retired | **-** | **-** |  | **-** | **-** |  | **-** | **-** |
| Others | **0.689 (0.239, 1.986)** | **0.490** |  | **-** | **-** |  | **-** | **-** |

Table S3 Odds ratios (OR) and 95% confidence intervals of having basic EHL stratified by education in the enter logistic regression

| **Risk factor** | **Junior high school and below** | |  | **Senior high school/vocational/ technical secondary school** | |  | **Junior college/bachelor degree and above** | |
| --- | --- | --- | --- | --- | --- | --- | --- | --- |
|  | **OR (95% CI)** | ***P*** |  | **OR (95% CI)** | ***P*** |  | **OR (95% CI)** | ***P*** |
| Gender |  |  |  |  |  |  |  |  |
| Male | Reference |  |  | Reference |  |  | Reference |  |
| Female | **0.335 (0.127, 0.884)** | **<0.05^*^** |  | **1.093 (0.569, 2.099)** | **0.790** |  | **0.632 (0.404, 0.988)** | **<0.05^*^** |
| Age groups |  |  |  |  |  |  |  |  |
| 15-34 | Reference |  |  | Reference |  |  | Reference |  |
| 35-49 | **0.329 (0.133, 0.818)** | **<0.05^*^** |  | **0.395 (0.174, 0.898)** | **<0.05^*^** |  | **0.109 (0.025, 0.483)** | **<0.01^**^** |
| 50-69 | **0.946 (0.570, 1.572)** | **0.831** |  | **1.057 (0.657, 1.699)** | **0.820** |  | **0.926 (0.632, 1.356)** | **0.692** |
| Region |  |  |  |  |  |  |  |  |
| Urban | Reference |  |  | Reference |  |  | Reference |  |
| Rural | **1.454 (0.852, 2.479)** | **0.170** |  | **1.099 (0.692, 1.746)** | **0.690** |  | **0.823 (0.550, 1.230)** | **0.341** |
| Occupation |  |  |  |  |  |  |  |  |
| Urban workers | Reference |  |  | Reference |  |  | Reference |  |
| Teacher | **14.834 (2.294, 95.923)** | **<0.01^**^** |  | **-** | **-** |  | **0.843 (0.399, 1.779)** | **0.654** |
| Leading cadres and civil servants | **1.993 (0.749, 5.306)** | **0.167** |  | **3.247 (0.961, 10.975)** | **0.058** |  | **2.428 (1.365, 4.318)** | **<0.01^**^** |
| Peasantry | **4.262 (1.341, 13.547)** | **<0.05^*^** |  | **1.125 (0.601, 2.108)** | **0.712** |  | **0.748 (0.392, 1.430)** | **0.380** |
| Student | **-** | **-** |  | **2.731 (1.313, 5.681)** | **<0.01^**^** |  | **1.410 (0.813, 2.445)** | **0.222** |
| The emeritus and retired | **-** | **-** |  | **-** | **-** |  | **2.490 (0.301, 20.564)** | **0.397** |
| Others | **-** | **-** |  | **0.699 (0.220, 2.217)** | **0.543** |  | **1.223 (0.479, 3.124)** | **0.673** |
